# Zoospore exudates from *Phytophthora nicotianae* affect immune responses in *Arabidopsis*

**DOI:** 10.1371/journal.pone.0180523

**Published:** 2017-06-29

**Authors:** Ping Kong, John M. McDowell, Chuanxue Hong

**Affiliations:** 1 Hampton Roads Agricultural Research and Extension Center, Virginia Tech, Virginia Beach, Virginia, United States of America; 2 Department of Plant Pathology, Physiology and Weed Science, Virginia Tech, Blacksburg, Virginia, United States of America; Fujian Agriculture and Forestry University, CHINA

## Abstract

Zoospore exudates play important roles in promoting zoospore communication, homing and germination during plant infection by *Phytophthora*. However, it is not clear whether exudates affect plant immunity. Zoospore-free fluid (ZFF) and zoospores of *P*. *nicotianae* were investigated comparatively for effects on resistance of *Arabidopsis thaliana* Col-0 and mutants that affect signaling mediated by salicylic acid (SA) and jasmonic acid (JA): *eds16* (enhanced disease susceptibility16), *pad4* (phytoalexin deficient4), and *npr1* (nonexpressor of pathogenesis-related genes1). Col-0 attracted more zoospores and had severe tissue damage when flooded with a zoospore suspension in ZFF. Mutants treated with ZFF alone developed disease symptoms similar to those inoculated with zoospores and requirements of EDS16 and PAD4 for plant responses to zoospores and the exudates was apparent. Zoospore and ZFFs also induced expression of the *PR1* and *PDF1*.*2* marker genes for defense regulated by SA and JA, respectively. However, ZFF affected more JA defense signaling, down regulating *PR*1 when SA signaling or synthesis is deficient, which may be responsible for *Arabidopsis* mutant plants more susceptible to infection by high concentration of *P*. *nicotianae* zoospores. These results suggest that zoospore exudates can function as virulence factors and inducers of plant immune responses during plant infection by *Phytophthora*.

## Introduction

*Phytophthora* species use a variety of strategies to infect plants, causing severe losses for food crops and ornamental plant production, as well as damage in forest ecosystems [1]. For example, *P*. *nicotianae* (syn. *P*. *parasitica*) can colonize foliar structures and roots of over 200 plant species including *Arabidopsis* [1–4]. This pathogen can use exudates from zoospores and bacteria to promote successful infection [3, 5, 6]. The exudates from zoospores of *Phytophthora* and *Pythium*, termed zoospore-free fluids (ZFF), function as quorum-sensing signals promoting zoospore homing and triggering infection by single zoospores [3, 5, 7]. However, it is not clear whether ZFF also affects plant immunity.

Plant immune responses are activated by two major classes of surveillance proteins: transmembrane pattern recognition receptors (PRRs) and plant nucleotide-binding, leucine-rich repeat (NLR) proteins [8, 9]. PRRs recognize conserved, pathogen-associated molecular patterns (PAMPS) to activate PAMP-triggered immunity (PTI). NLR proteins recognize pathogen effectors to activate effector-triggered immunity (ETI). Activation of PTI and ETI in plants involves a complex signaling network regulated by small plant hormone molecules such as salicylic acid (SA), jasmonic acid (JA) and ethylene (ET), as well as proteins responsible for production and signal transduction of these hormones [10, 11].

*Arabidopsis thaliana* is a popular model plant for studying plant immunity and there are many loss-of-function *Arabidopsis* mutants available for dissection of immune responses to different pathogens. For example, *eds* 16–1 contains a loss of function mutation in a gene encoding isochorismate synthase, a major enzyme in the biosynthesis [12] of salicylic acid (SA), which plays a central role in immune responses to biotrophic pathogens. Similarly, *pad*4-1 is a mutant of the Phytoalexin Deficiency 4 gene that encodes a lipase-like protein operating upstream of SA accumulation to regulate various aspects of immune responses, including production of the anti-microbial phytoalexin camalexin, and SA-dependent immune signaling, and other sectors of the immune response network [13–16]. *Npr-1* is a mutant of Nonexpresser of Pathogenesis-Related Protein 1 (NPR1), encoding a receptor of SA and a central regulatory protein of SA-mediated responses in plant defense [11, 17, 18]. Another useful tool in *Arabidopsis* is marker genes that are assayed to report activation of specific defense signaling pathways. For example, *PR1* [19] and *PDF1*.*2* [20] are widely used to determine activation of pathways mediated by SA and JA/ET signaling, respectively.

Current models of defense signaling indicate that SA and JA/ET pathways are activated by pathogens with different lifestyles [21]. For example, activation of JA/ET signaling is often associated with the defense against necrotrophic such as *Pythium* species [22–25]. In contrast, activation of SA signaling is a key component of defense against biotrophs such as *Hyaloperonospora arabidopsidis* [26–28]. The SA and JA/ET pathways are mutually antagonistic in *Arabidopsis*, such that activation of one pathway triggers repression of the other. It is believed that this mutual antagonism provides for adaptive tailoring so that the most effective defense is deployed against the type of pathogen that is invading the plant [29]. However, it is unclear how these pathways are regulated during infection by hemi-biotrophic pathogens that colonize the plants as biotrophs and later activate a necrotrophic phase.

Many *Arabidopsis*-based pathosystems have been studied for plant defense mechanisms against oomycetes of importance, including *Hyaloperonospora arabidopsidis*, *Phytophthora capsici*, *P*. *cinammomi*, *P*. *infestans*, *P*. *nicotianae*, *P*. *porri*, *P*. *sojae*, and *Pythium irregulare* [22, 28, 30–34]. However, only *H*. *arabidopsidis* is a natural pathogen of *Arabidopsis*, while *P*. *porri* is a natural pathogen of *Brassica* plants related to *Arabidopsis*. Most of other oomycetes either cause a hypersensitive response in *Arabidopsis* under controlled conditions or have a restricted host range [2, 24, 31, 35–38]. In contrast, *P*. *nicotianae* has a wide host range causing diseases of over 200 plant species and can infect *Arabidopsis* at a high inoculum concentration in the laboratory [2, 3]. Moreover, *P*. *nicotianae* is relatively amenable to biological, molecular and genetic approaches. Thus, the *Arabidopsis*—*P*. *nicotianae* phytopathosystem is considered as a useful model for studying interaction between plants and oomycete pathogens.

In this study, we compared how *P*. *nicotianae* zoospores and ZFF interacted with *A*. *thaliana* Col-0 and mutants in SA signaling and biosynthesis. While previous studies focused on roles of ZFF in *Phytophthora* quorum sensing and behaviors, this study focused on the impact of zoospore exudates on plant immune responses during infection by *P*. *nicotianae*.

## Materials and methods

### Pathogen growth conditions and ZFF preparation

*Phytophthora nicotianae* isolate 1B11 from annual vinca (*Catharanthus roseus* cv. ‘Little Bright Eye’) was used in this study. Growth, maintenance and preparation of zoospore-free fluid (ZFF) of the organism followed previous protocols unless otherwise stated [3, 5, 39]. Specifically, high concentrations of zoospore suspensions were made by growing mycelium plugs in 10% clear V8 juice broth at 23°C for one week in the dark followed by inducting sporangia under the lights for two days, rinsing and flooding of the grown mycelium/sporangium mats with cold sterilized distilled water (SDW) to remove the nutrient residues from media and to release zoospores, respectively. Zoospore suspensions were measured for concentrations with a hemocytometer under a microscope. To make ZFF, suspensions were filtered through a 0.2 μm filter after vortexed for 5 min to allow zoospore to encyst and release chemicals into the solution. The filtrate was referred as ZFF, and its concentration was determined by the concentration of zoospores suspension used. In this study, all ZFF were from zoospore suspensions at 10^5^ /ml or higher.

### Plant growth conditions

*Arabidopsis thaliana* (Col-0) and mutants, *eds*16-1, *npr*1-1 and *pad*4-, were seeded in Metro Mix 360 top-layered with Metro Mix 200 (Scotts, Marysville, OH) and grown for 5 weeks in a growth chamber at 25°C with a 14/10 h light/dark cycle. All plants were fertilized in the third and fourth week with 20-20-20 liquid fertilizer (Scotts, Marysville, OH). Four- to 6-week-old plants were used in assays including microscopy, disease assessment and gene expression.

### Examination of effects of ZFF on plant response to zoospores

Detached cauline leaves from 5-week old Col-0 plants were placed individually in the wells of a 24-well plate and flooded with 250 μl of ZFF or sterile distilled water (SDW), each containing zoospores at a concentration of 1,600/ml. The plate was kept in the dark overnight at 23°C, and then transferred to growth chamber. Leaves in the wells were examined for zoospore aggregation and plant tissue damage under a Nikon Labophot-2 or Olympus 1X71 inverted microscope after 5, 24, 48 and 120 h incubation. Each treatment (ZFF or SDW) included three replicated wells.

### Assessment of effects of ZFF on disease development

Plants of four to five weeks were removed from MetroMix, cleaned in tap water and rinsed with SDW before treatment. Six plants per genotype were flooded with 20 ml ZFF or controls SDW and zoospore suspension at 200,000/ml, respectively in a 40-ml beaker and placed in moisture containers. Plants were kept at 23°C in the dark for 16 h and then placed in a growth chamber under the plant growth conditions. Disease was rated by the percentage of symptomatic leaves in total leaves at 88 h after treatment. Yellowing and rotting symptoms were rated separately. Each experiment included four biological replicates and was repeated once.

### Analysis of effects of ZFF on plant gene expression

ZFF was also assessed for activation of SA and JA signaling mediated defense marker genes *PR*1 and *PDF*1.2 in a time course experiment. The experiment was set up as that for disease assessment and included three replicates for each treatment: ZFF, SDW or zoospores at 200,000/ml. At 4, 8, 16, 24, 48 and 72 hours after treatment, one plant was sampled from each replicate. Replicate plants were combined and extracted for RNA with the RNeasy Mini Kit and RNase-Free DNase Set (Qiagen, Valencia, CA). Equal amounts of RNA were used to perform qRT-PCR in the ABI 7500 Real Time PCR System with the High Capacity cDNA Archive Kit (Applied Biosystems, Foster City, CA), Power SYBR^®^ Green PCR Master Mix (Life Technologies, Carlsbad, CA) and primers for target genes *PDF*1.2 [40] and *PR*1 [41] as well as for the endogenous control, reference gene Actin-1 (*ACT*1) [40]. All reactions were run at the 9600 mode Emulation with 50°C for 2 min at first stage, 95°C for 10 min at second stage followed by 40 cycles of 15 sec at 95°C, 1 min at 60°C at the third stage. Each sample had three PCR runs for each of the genes. The mean value of Ct (threshold cycle) of each gene in a sample of the PCRs were generated with Relative Quantification Study in the software SDS v.1.3.1 (Applied Biosystems, Foster City, CA). Target gene expression was measured with Relative transcription levels (RQ) which was calculated using the formula 2^-dCt^ after Ct of target gene was normalized with that of the reference gene *ACT*1 in the same sample (dCt_target_ = Ct_target_−Ct_*ACT*1_).

### Statistical analysis

Standard errors were calculated from standard deviation and number of replicates with Microsoft Excel. T-test at equal variances in Excel was used for evaluating statistical significance between the treatments. *P* values <0.05 were considered significant.

## Results

### Effect of zoospore exudates on *Arabidopsis* infection by *Phytophthora nicotianae*

**Fig 1:**
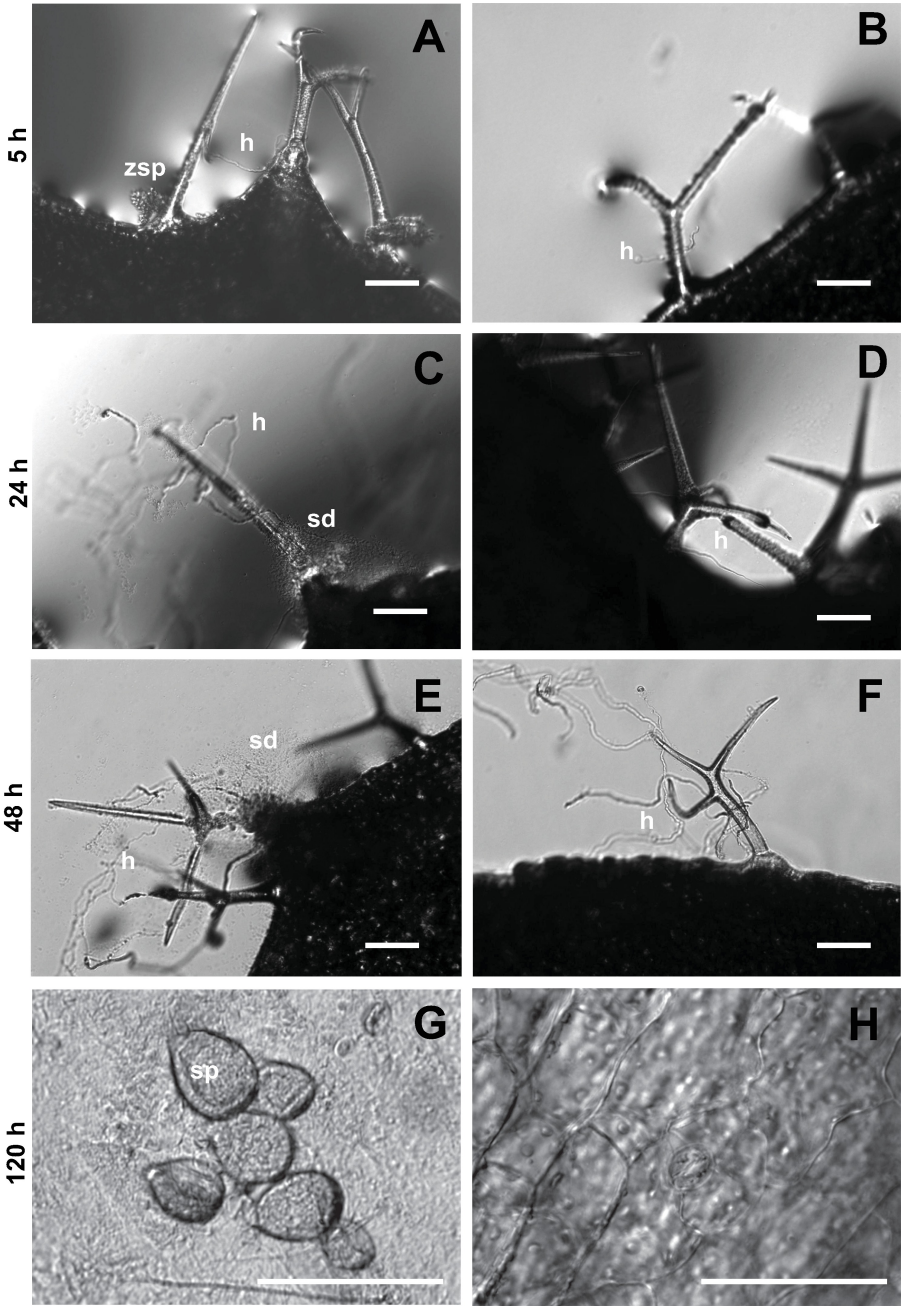
Microscopy of effects of zoospore free fluid (ZFF) on Arabidopsis (Col-0) responses to *Phytophthora nicotianae* zoospores. Individual cauline leaves were flooded in ZFF (A, C, E, and G) or sterile distilled water (SDW) (B, D, F and H) containing zoospores at 1,600 /ml and observed at different exposure time. Bars = 50 μm. Letters: h = hypha or hyphae, sd = substance discharge from plant tissues, sp = sporangia, zsp = zoospores.

Detached cauline leaves of Col-0 were flooded in ZFF containing zoospores at 1, 600/ml for 5 h (Fig 1A). At this time point, zoospores aggregated at the base of trichome. The affected trichomes became macerated 24 h after the exposure. Meanwhile, there was zoospore germination and mycelium growth on and in plant tissues (Fig 1C). Leaf tissue damage and substance discharge from trichomes was evident at 48 hours after the treatment (Fig 1E). Leaf yellowing was also evident at this time point. On the 5th day of the treatment, rotting became apparent and further tissue damage and production of numerous sporangia was observed under the microscope (Fig 1G). Same effect was found for ZFF from zoospore suspensions at ≥ 10^5^/ml as used in this study. In contrast, there was sparse zoospore attachment to leaves treated with SDW (Fig 1B). SDW-treated leaves/sustained little damage in trichomes or leaf tissue and supported limited hyphal growth with no sporangia production (Fig 1B, 1D, 1F and 1H). These results indicate that ZFF promoted susceptibility of *Arabidopsis* plants to *P*. *nicotianae*.

### Effect of zoospore exudates on *Arabidopsis* defense components

**Fig 2:**
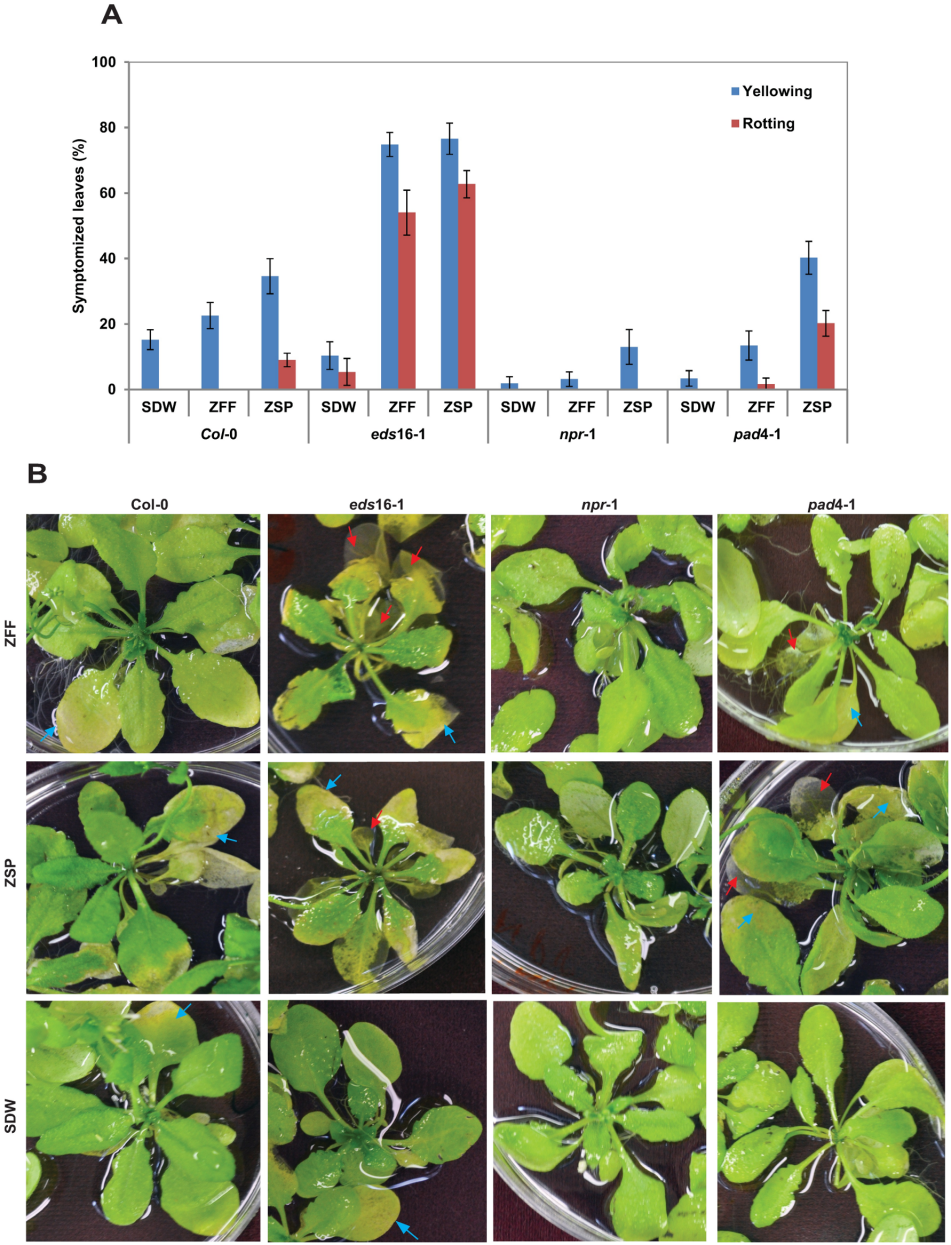
Comparison of effects on disease symptom development of Arabidopsis Col-0 and its mutant *eds*16-1, *npr*1-1 and *pad*4-1 between zoospore free fluid (ZFF) and zoospores (ZSP) of *Phytophthora nicotianae*. Plants were treated with ZFF alone or zoospore suspension at 200,000/ml and recorded symptoms after 88 h at 23°C. A. Percentage of leaves displaying yellowing or rotting after treatment. Each column is a mean of 6 plants; bars depict standard error. Similar results were observed in three independent replicates. B. Images of the plants after treatment. Typical yellowing or rotting is indicated with blue and red arrows, respectively.

To better understand why plants become more susceptible to zoospores with addition of ZFF, *Arabidopsis* Col-0 and mutant plants *eds*16-1; *npr*1-1 and *pad4*-1(all in the Col-0 background) were treated with a zoospore suspension at 200,000/ml; ZFF alone, or a SDW control (Fig 2, Table 1). Visual symptoms were compared in response to all three treatments. Zoospore treatment induced visible symptoms in >40% of leaves from Col-0. Contrastingly, ZFF treatment induced only a minor increase in symptoms compared to water-treated control. Interestingly, *eds*16-1 plants developed severe leaf yellowing and rotting, compared to Col-0, after treatment with ZFF (Fig 2B). Similar symptoms were displayed after inoculation with zoospores. The *pad* 4–1 mutant also displayed slightly enhanced symptoms, compared to wild-type Col-0, after treatment with ZFF or zoospore inoculum. Interestingly, responses to ZFF and zoospores were slightly attenuated in *npr*1-1 compared to Col-0. These results indicate that a deficiency in SA biosynthesis/signaling rendered *Arabidopsis* more susceptible to *P*. *nicotianae*, and that ZFF treatment triggered disease-like symptoms in an SA-deficient mutant.

**Table 1:** Difference of effects between zoospore free fluid (ZFF) and zoospores (ZSP) of *P*. *nicotianae* on symptom production of *Arabidopsis thaliana* plants.

Plant	*P* value between treatments ^X^					
	Yellowing			Rotting		
	ZFF:ZSP	ZFF:SDW	ZSP:SDW	ZFF:ZSP	ZFF:SDW	ZSP:SDW
Col-0	**0.041**	**0.004**	1.597	**0.003**	NA^Y^	**0.003**
*eds*16-1	0.376	**<0.001**	**<0.001**	0.134	**<0.001**	**<0.001**
*npr*1-1	**0.046**	**0.029**	0.319	NA	NA	NA
*pad*4-1	**<0.001**	**0.027**	**<0.001**	**<0.001**	0.170	**<0.001**

^X^ *P* value is determined with t-Test with equal variance in Excel at α = 0.05. A difference at *P* ≥ 0.05 is not significant.

^Y^ NA = not applicable.

### Effect of zoospore exudates on regulation of SA and JA signaling in the plants

**Fig 3:**
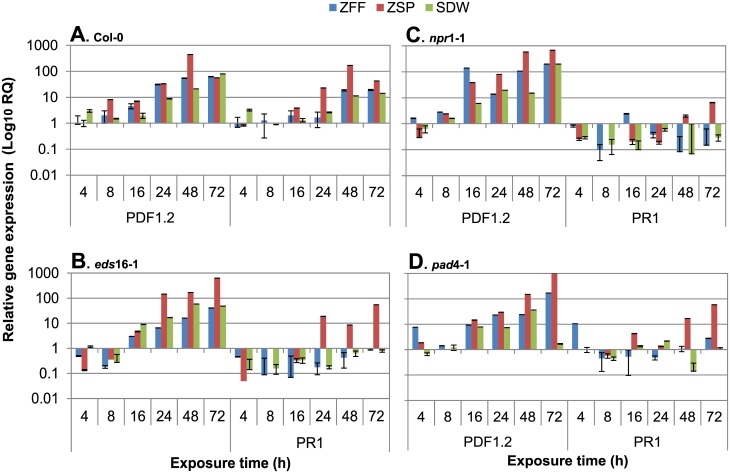
Effects of zoospore free fluid (ZFF) of *Phytophthora nicotianae* on expression of salicylic acid (SA)—and jasmonic acid (JA)—associated defense genes in Arabidopsis Col-0 and its mutants. Plants were flooded with ZFF, SDW or zoospore suspension at 200,000/ml (ZSP) and extracted for RNA at 4, 8, 16, 24 and 48 h after treatment. *PR1*, *PDF1*.*2* expression was analyzed with QRT-PCR in a real time PCR system and the transcript levels or mean value of Ct (threshold cycle) were normalized with reference gene *Actin-1* in the same samples. The graph depicts the relative expression of the genes in treated plants. Each column is a mean of three PCR replicates from one of two biological experiments with similar results. Bars depict standard error from the replicates.

To determine how ZFF may affect defense signaling pathways, expression of SA and JA marker gene *PR1* and *PDF1*.*2* was examined in wild type and mutant plants treated with zoospores, ZFF, or SDW. In Col-0, both genes were induced by ZFF and zoospores although the effect of ZFF was weaker than zoospores. ZFF treatment appeared to have a stronger effect on *PDF1*.*2* expression than on *PR1* expression, compared to the water treatment (Fig 3A). *eds*16-1 plants displayed substantially different patterns of transcript accumulation, compared to Col-0: In zoospore- and ZFF-treated plants, expression of *PDF1*.*2* was much lower at 4 and 8 hours after treatment, compared to Col-0, but then increased to a level higher than Col-0 by 72 hours. Zoospore-responsive expression of *PR-1* was lower than Col-0 in *eds*16-1 at early time points, but increased at later time points. As in Col-0, ZFF effect on both marker genes in *eds*16-1 was roughly equivalent to that of water. The *npr1-1* and *pad4-1* mutants were similar to each other and to *eds16-1*: Expression of *PDF1*.*2* was equivalent or somewhat higher to Col-0, with zoospore treatment inducing stronger expression than ZFF or water. *PR-1* expression in mutants was much lower than in Col-0 in response to all treatments although zoospore treatment induced a much stronger response in the late time.

## Discussion

Zoospore exudates of *Phytophthora* have been shown as chemical signals for *Phytophthora* quorum sensing leading to zoospore aggregation, homing and infection initiation as well as communications between zoosporic oomycetes and with bacteria [3, 5, 7, 39, 42]. This study reveals new roles of zoospore exudates as virulence factors of the pathogen and inducers of plant immunity, contributing to the success of plant infection by *Phytophthora* zoospores.

Most importantly, zoospore pre-treatment with ZFF rendered *Arabidopsis* plants more susceptible to infection by *P*. *nicotianae* zoospores (Fig 1). Additionally, ZFF treatment induced disease-like symptoms of yellowing and rotting that were similar to those induced by treatment with zoospores. Finally, ZFF treatment influenced expression of marker genes for both SA- and JA-dependent immune responses. These data indicate that zoospore exudates can affect the function of the host immune system, in addition to mediating intra- and interspecies chemical communication, promoting zoospore homing and plant infection described in previous studies. The ability of ZFF to promote disease-like symptoms, independent of pathogen colonization, is particularly intriguing. We propose that the chlorosis and rotting are due to one or more toxins present in ZFF. It would be of great interest to identify the precise compounds in ZFF that induces these symptoms. It will also be interesting to determine if the toxins promote immune activation as part of the necrotrophic phase of plant colonization.

Another interesting aspect is that plant responses to zoospores and ZFF treatment appear linked to SA signaling, based on differential responses observed during treatment of mutants that disable SA biosynthesis (*eds16-1*) or signaling (*pad4-1*, *npr1-1*). When SA biosynthesis is disabled (*eds*16-1), the disease symptoms triggered by zoospores or ZFF are substantially enhanced. A similar but weaker enhancement was observed in the *pad4-1* mutant. These data indicate that SA signaling plays a role in resistance to pathogen colonization (zoospores) and/or toxicity mediated by ZFF. Because *pad4-1* was less responsive to ZFF, PAD4 may partially involve the resistance to the toxins in zoospore exudates despite the fact that it can provide amplified defense through phytoalexin camalexin production or interaction with EDS [14, 15]. Interestingly, *npr1-1* mutants displayed reduced symptoms to both treatments, suggesting that the resistance to *P*. *nicotianae* and ZFF-induced disease symptoms occurs through a mechanism that requires SA and PAD4, but operates independently of NPR1. Although *npr1-1* has been associated with SA signaling in many interactions [43, 44] including the oomycete *Hyaloperonospora arabidopsidis* [11], genetic studies have provided evidence for immune responses that are SA-dependent but to not require NPR1 [45–48].

The responsiveness of *PR-1* and *PDF*1.2 to *P*. *nicotianae* zoospores indicates that SA and JA pathways are responsive, in *Arabidopsis*, to *Phytophthora* pathogens [2, 31]. Interestingly, ZFF treatment had a more pronounced effect on *PDF*1.2 expression than on *PR*-1 expression, suggesting that a component of ZFF was sufficient to trigger JA-dependent immune responses (Fig 3). It is conceivable that ZFF may be more involved in necrotrophic activities, or that activation of JA defenses may function to suppress SA defenses during biotrophic growth. This is apparent when SA function is attenuated; ZFF induced yellowing and rotting on the treated plants while induced no or very low *PR*-1 response in the mutants, and high concentration of zoospores invoked much more potent responses on defense signaling and symptoms (Figs 2 and 3B–3D). This regulatory feature of ZFF on plant defense may explain why addition of ZFF in a low concentration of zoospore inoculum which is technically equivalent to high concentration of zoospores in the term of the impact on quorum sensing mediated infection [3], enhanced susceptibility of plants (Fig 1). Similar complex responses have been well documented for necrotrophs with a broad host range which can produce diverse PAMPs that activate plant immune responses as well as virulence factors that suppress immune responses [49, 50]. Understanding the exact molecular mechanisms that drive these responses will be a significant step towards understanding the basis of resistance and susceptibility to necrotrophic and hemi-biotrophic pathogens that cause substantial crop loss.

Using an external control is uncommon in a time course study on plant–pathogen interaction with qRT-PCR. In this study, we used SDW as the control to compare zoospore inocula and exudates at the same condition. However, SDW treatment caused responses of SA and JA defense signaling marker gene which sometimes was equivalent to those triggered by zoospores or ZFF, indicating that flooding may induce similar SA or JA responses. Net effects of zoospores and ZFF can be obtained by calibrating transcripts of these target genes from the same sampling time in the individual treatments with transcripts of the control. Alternatively, calibration may be done with samples at zero exposure time. However, reduction of such background may cover up real effects of zoospore inoculum or exudates on plant defense which gave rise to visual symptoms distinct from that of SDW (Fig 2). Nevertheless, to include external control in qRT-PCR for analyses of plant defense gene expression, it is warranted to use SA and JA marker genes that are not responsive to flood or to develop alternative feasible inoculation or treatment methods.

## Supporting information

S1 Table Ct (threshold cycle) and RQ values of tested genes in qRT- PCR. Data were generated in ABI 7500 Real Time PCR System and computed with Relative Quantification Study in the software SDS v.1.3.1.
(XLSX)

S2 Table Rated Arabidopsis leaves that showed yellowing or rotting in treated plants after 88 h treatment at 23°C with ZFF and controls of sterile distilled water (SDW) and high concentration of zoospores at 200,000/ml. (XLSX)
